# Supplementary material for: Identification and characterization of the CRK gene family in the wheat genome and analysis of their expression profile in response to high temperature-induced male sterility
Source: PeerJ. 2024 May 7;12:e17370. doi: 10.7717/peerj.17370 (PMC11086307; doi:10.7717/peerj.17370)
Supplement: Supplemental Information 1 [file peerj-12-17370-s001.pptx]

## Slide 1
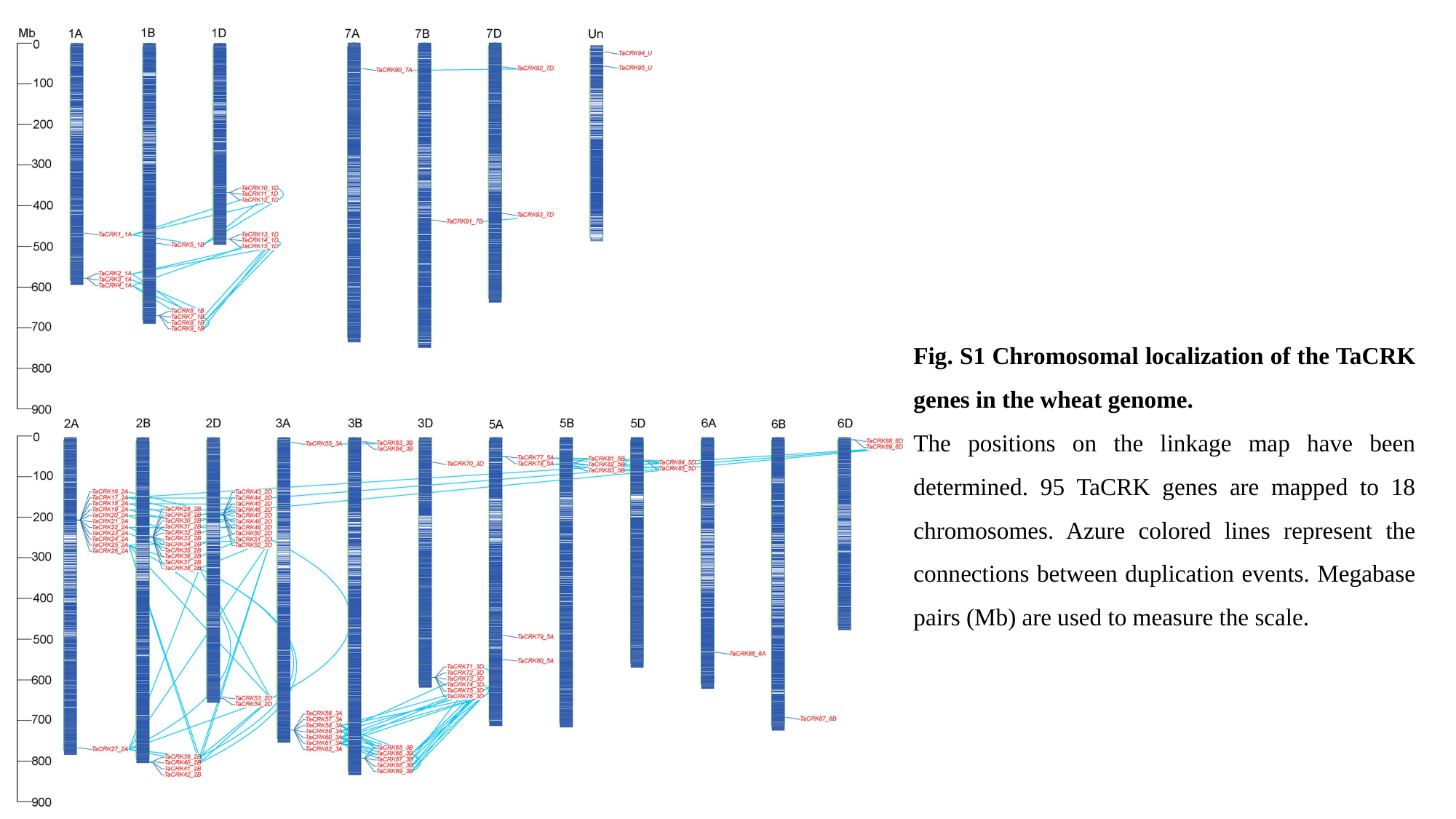

Fig. S1 Chromosomal localization of the TaCRK genes in the wheat genome.
The positions on the linkage map have been determined. 95 TaCRK genes are mapped to 18 chromosomes. Azure colored lines represent the connections between duplication events. Megabase pairs (Mb) are used to measure the scale.

## Slide 2
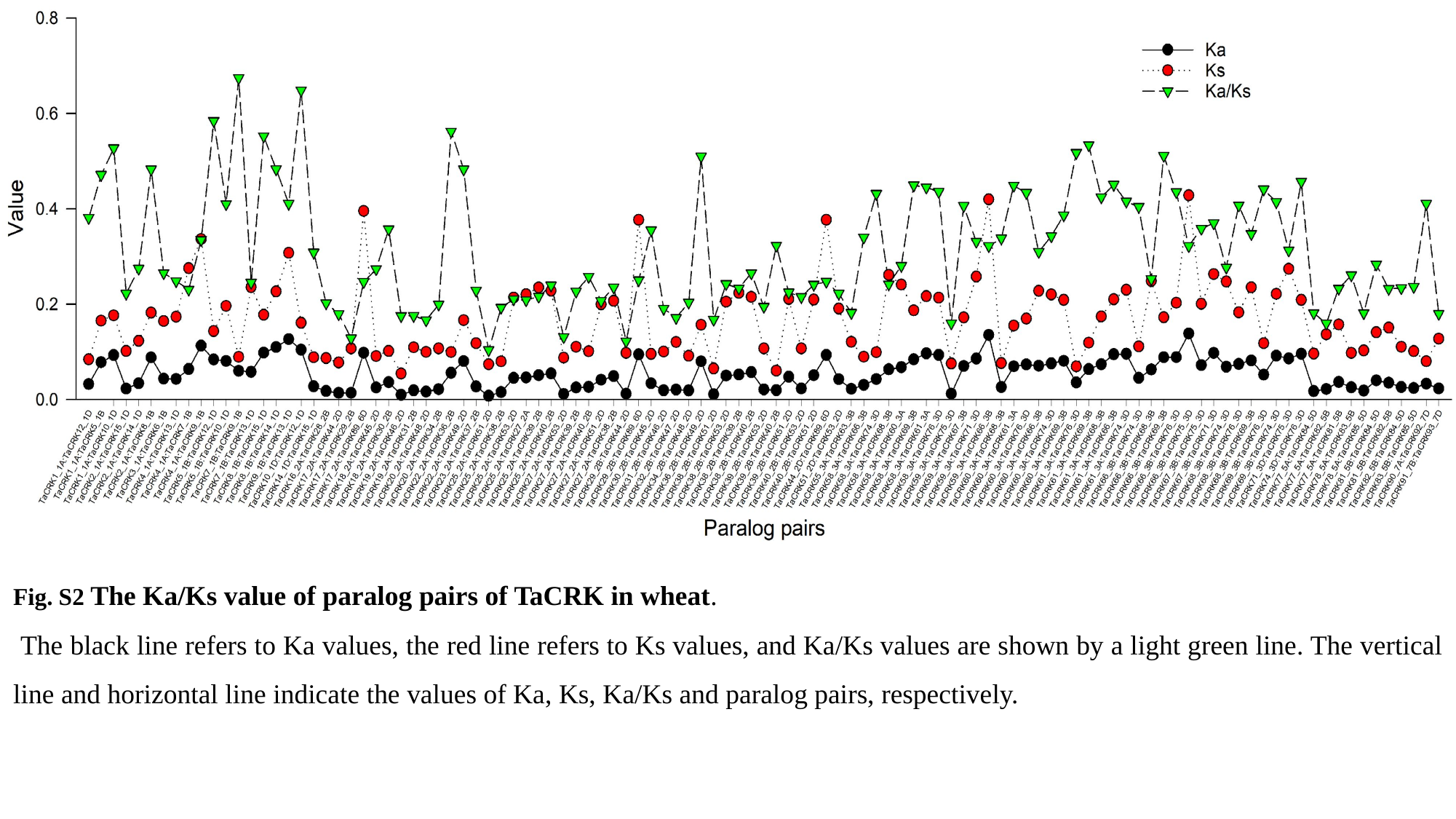

Fig. S2 The Ka/Ks value of paralog pairs of TaCRK in wheat.
 The black line refers to Ka values, the red line refers to Ks values, and Ka/Ks values are shown by a light green line. The vertical line and horizontal line indicate the values of Ka, Ks, Ka/Ks and paralog pairs, respectively.

## Slide 3
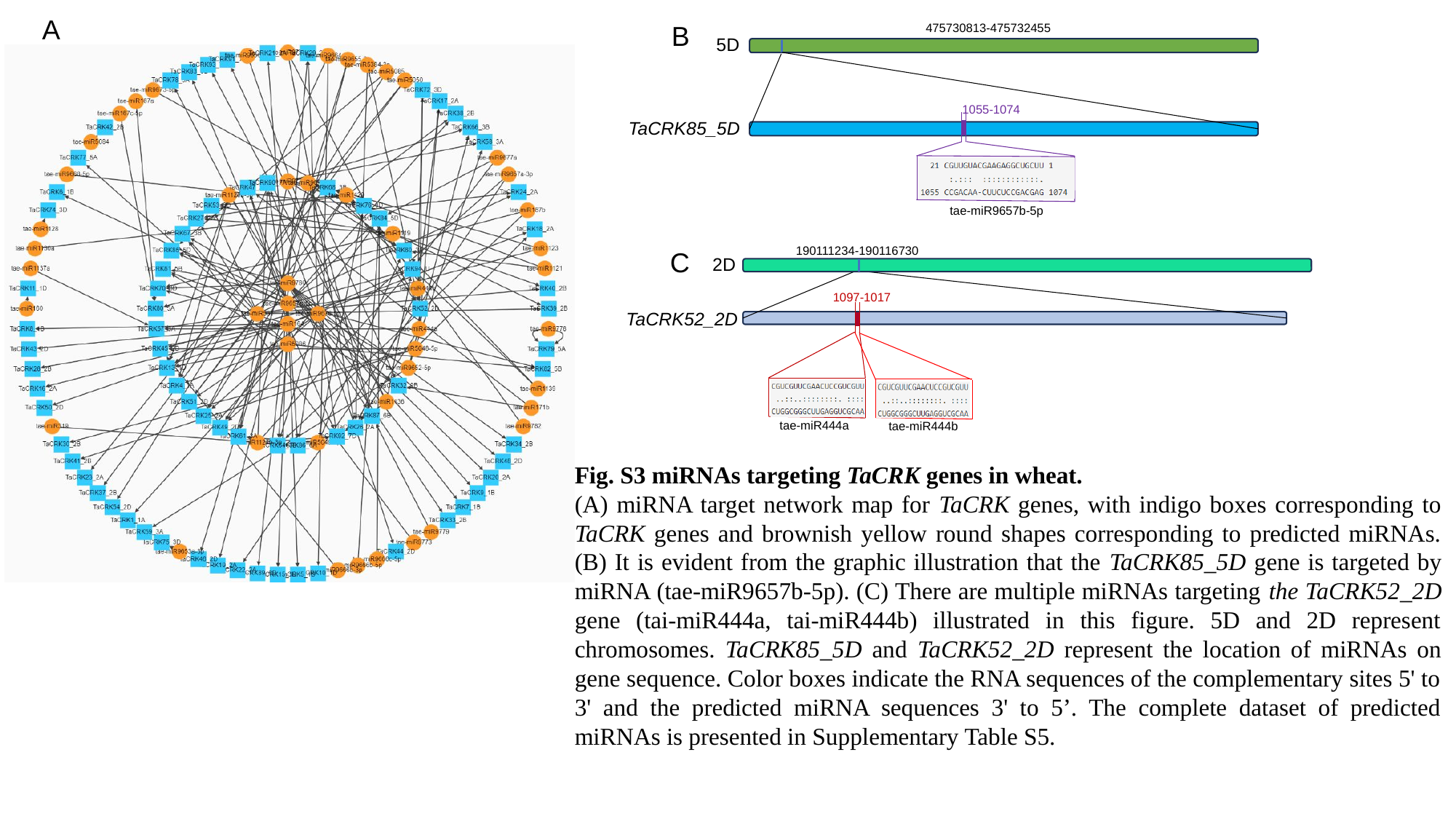

A
B
475730813-475732455
5D
1055-1074
TaCRK85_5D
tae-miR9657b-5p
190111234-190116730
C
2D
1097-1017
TaCRK52_2D
tae-miR444a
tae-miR444b
Fig. S3 miRNAs targeting TaCRK genes in wheat.
(A) miRNA target network map for TaCRK genes, with indigo boxes corresponding to TaCRK genes and brownish yellow round shapes corresponding to predicted miRNAs. (B) It is evident from the graphic illustration that the TaCRK85_5D gene is targeted by miRNA (tae-miR9657b-5p). (C) There are multiple miRNAs targeting the TaCRK52_2D gene (tai-miR444a, tai-miR444b) illustrated in this figure. 5D and 2D represent chromosomes. TaCRK85_5D and TaCRK52_2D represent the location of miRNAs on gene sequence. Color boxes indicate the RNA sequences of the complementary sites 5' to 3' and the predicted miRNA sequences 3' to 5’. The complete dataset of predicted miRNAs is presented in Supplementary Table S5.
